# Might Gendering Ski Binding Settings be Helpful for the Prevention of ACL Injuries Among Female Recreational Alpine Skiers?

**DOI:** 10.1186/s40798-022-00415-0

**Published:** 2022-02-05

**Authors:** Markus Posch, Gerhard Ruedl, Katja Tecklenburg, Martin Burtscher

**Affiliations:** 1 Department of Sport Science, University of Innsbruck, Fürstenweg 185, 6020 Innsbruck, Austria; 2 medalp sportclinic, 6460 Imst, Austria

**Keywords:** Gender, Sex, Alpine skiing, Knee injuries, Ski binding

## Abstract

When interpreting sex differences in the injury risk during sport activities, potential gender effects are often overlooked. This might actually be the case with regard to the higher injury risk of the anterior cruciate ligament (ACL) in female skiers. A higher failure rate of ski binding releases has been suggested at least partly to explain the more frequent ACL injuries in female skiers. However, as males seem to be predominantly responsible for the development of standards for ski binding settings, one might speculate that they could rather make standards for males than females. If true, the inclusion of female engineers could actually represent an appropriate approach to reduce ACL injures in female recreational skiers.

## Key Points

- There is still a need to distinguish clearly between sex and gender when interpreting sex differences in injury risk during sport activities.
- Failure of binding release at the moment of a ski accident may contribute to the higher injury risk of the ACL in female skiers.
- When males are predominantly responsible for the development of standards for ski binding settings, they might partly neglect actual needs for females.

## Background

Female recreational alpine skiers have twice the knee injury prevalence of their male counterparts, and the ACL injury risk is three times greater in females.

## Sex Versus Gender Effects

There is still an increasing necessity to entangle sex and gender in human health research when interpreting sex differences in the prevalence and incidence of various diseases and injuries [1]. The successful entanglement of sex and gender requires a serious and systematic assessment of the complex interaction between biology and the social environment across all ranges of our society. This interaction starts shortly after birth and remains of clinical and social relevance throughout all stages of life. However, the role of sex as a social construct is conspicuously missing [2]. Gender effects on various health- and disease-related issues have been highlighted in the literature, e.g., for cardiovascular diseases [3], for autism spectrum disorders [4] but also for injuries of the ACL [2].

## Are Sex Differences in Skiing Injuries Related to Gender Effects?

Parsons et al. [2] suggest that embedding gender when researching ACL injury would heighten the awareness of possible influences outside of the traditional biological elements like hormonal status or anatomic characteristics. Considering such sex-specific factors could lead to better approaches in the prevention and treatment of ACL injuries [2]. This article made us rethink our observations of the twice as frequent knee injury rates in female recreational alpine skiers compared to their male counterparts [5]. We suggested that one reason may be the higher failure rate of ski binding releases in females due to an existing discrepancy between their muscle strength of the lower extremities and the release setting standards of ski bindings [5]. This assumption was based on an epidemiological study demonstrating that out of 1300 recreational skiers who had sustained a knee injury, only 32% of male skiers but 51% of female skiers reported failure of binding release at the moment of accident [5]. Another study by Posch et al. [6] also showed that uninjured recreational female skiers were three times more often unable to self-release their ski bindings compared to their male counterparts, although their bindings had been correctly adjusted according to the International Standards Organization (ISO) 11,088 standard for binding setting values. The ISO 11088 standard considers body mass, height, age category, ski boot sole length and self-estimated skier type but not a “sex” factor. Therefore, it has been suggested to include a correction factor by applying a reduction of release torques for female skiers. It could be already shown that such a reduction was associated with a lower risk for knee injury [7]. However, these findings mean an association and not explicit cause and effect relationship because at the same time Ettlinger et al. (2006) [8] showed a decrease in ACL injury risk without lowering binding settings.

## How to Explain Potential Gender Effects on the Rate of Knee Injuries in Female Skiers?

Could it be that the higher rate of knee injuries in female recreational alpine skiers is due to the fact that predominantly males have been responsible for the development of standards for ski binding settings? If yes, one could argue that males will rather develop standards for males, thereby neglecting actual needs for females. Thus, a sex-balanced composition of the standardization group would be highly desirable but may not always be the case. At the moment, there are only 4 females (11%) out of 35 members of the expert group that deals with the ISO 11088 standard for binding setting values, likely as a consequence of an existing “engineering gender gap”. This fact, whether it is socially acceptable or desirable for women to participate in various spheres of life, has already been discussed by Parsons et al. [2]. The existence and consequences of such an underrepresentation of women especially in the field of engineering have recently been emphasized by Peters [9] in a somewhat different context. Similar to the ski binding issue, Peters [9] concluded that “a male-dominated engineering profession has made critical errors, from cars that are safer for men than women to a dearth of women-sized space suits”. This example not only underlines the complexity by which biological conditions could be influenced by a variety of environmental factors, but also draws attention to the importance of identifying possible sex-related key factors or improved understanding and interpreting gender aspects of sex differences. We fully agree that the ACL injury cycle requires a holistic and intersectional approach [2].

## Conclusion

In conclusion, addressing sex-specific influence factors for alpine ski binding release, taking great account from female engineers, could actually represent an appropriate approach to reduce ACL injures in female recreational skiers and therefore have a valuable impact on women’s health, fitness and well-being.

## Data Availability

Not applicable.

## Abbreviations

ACL: Anterior Cruciate Ligament
ISO: International Standards Organization
